# Multifunctional Nd^3+^ substituted Na_0.5_Bi_0.5_TiO_3_ as lead-free ceramics with enhanced luminescence, ferroelectric and energy harvesting properties†

**DOI:** 10.1039/c8ra01349g

**Published:** 2018-04-23

**Authors:** Kumara Raja Kandula, Saket Asthana, Sai Santosh Kumar Raavi

**Affiliations:** Advanced Functional Materials Laboratory, Department of Physics, Indian Institute of Technology Hyderabad Kandi, Sangareddy 502285 Telangana India sskraavi@iith.ac.in asthanas@iith.ac.in

## Abstract

Herein, we present comprehensive investigations of the optical and electrical properties of Nd^3+^ substitution in sodium bismuth titanate ceramics (NBNT) with varying Nd^3+^ concentration. The room temperature photoluminescence (PL) emission for both unpoled and poled samples is observed to be a maximum for an Nd^3+^ substitution of 1 mol%. Upon poling, the PL intensity is observed to be quenched, consistent with the obtained XRD data, indicating an electric-field induced structural ordering towards higher symmetry, confirmed with the help of structural refinement. The evaluated ferroelectric to relaxor and antiferroelectric relaxor *T*_(F–R)_ was observed clearly from the poled dielectric–loss curve for the 1 mol% of Nd^3+^ substitution. Furthermore, the optimized NBNT exhibited a lower *E*_c_ and a higher off-resonance figure of merit (FOM_off_) for energy harvesting by 12% and 30%, respectively, in comparison with un-doped NBT.

## Abstract 2

Herein, we present investigations on the effect of electrical poling on the optical and electrical properties of Nd^3+^ substituted NBT ceramics with varying Nd^3+^ concentrations.

## Graphical abstract

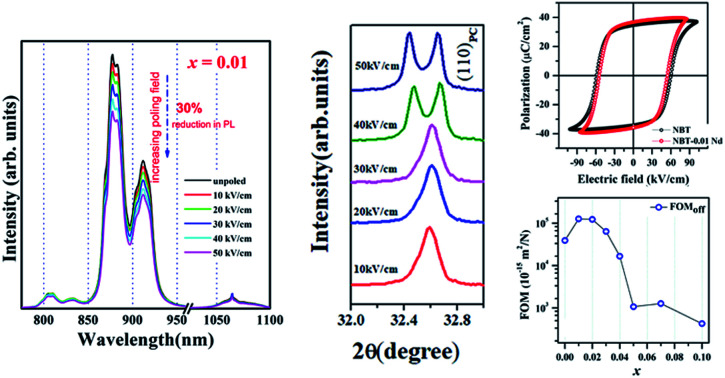

## 1. Introduction

Multifunctional ceramics, exhibiting two or more properties from among magnetic, ferroelectric, pyroelectric, piezoelectric and optoelectronic properties have received a remarkable amount of interest.^1–3^ Sodium bismuth titanate, Na_0.5_Bi_0.5_TiO_3_ (NBT), with a lead-free perovskite structure is a proven candidate showing excellent ferroelectric properties.^4,5^ Enhanced piezoelectric properties of NBT and its solid solutions by cation substitution at the A and B-sites have been reported.^6–9^ In particular, it was observed that site-specific substitution with optically active rare-earth (RE) ions resulted in enhanced electrical properties with the additional functionality of photoluminescence (PL), making them attractive for promising applications.^10–15^ Recent reports on RE substituted NBT ceramics indicated that both PL and electrical properties can be modified upon electrical poling.^16,17^ Among all the optically active RE ions, Nd^3+^ with emission in the near IR spectral region has found a plethora of optoelectronics applications. M. Zannen *et al.*^13^ reported the best emission together with better ferroelectric properties for 5 mol% substitution of Nd^3+^ for NBT ceramics. Another investigation^18^ revealed better piezoelectric coefficients with 2 mol% substitution of Nd^3+^. These contradictory reports, however, did not deliberate on the effect of electrical poling.

In this report, we focus our attention on obtaining the optimal substitution of Nd^3+^ for enhanced properties and multifunctionality. Here, we relate the role of structural changes in the host lattice to the obtained PL intensity from Nd^3+^ substituted [Na_0.5_Bi_0.5−*x*_Nd_*x*_]TiO_3_ (referred to hereafter as NBNT) ceramics with varying Nd^3+^ concentration in the range of *x* = 0.003–0.1 [≅(0.3–10) mol%] by photo excitation at 532 nm. We observed the maximum PL intensity for *x* = 0.01 (≅1 mol%), which is the critical concentration for the onset of PL quenching. Upon poling, the applied electric-field induces structural order that is manifested in further PL quenching. The obtained hysteresis (*P*–*E*) data for NBNT showed a reduced coercive electric field (*E*_c_) with almost identical remnant-polarization (*P*_r_), implying that optimized NBNT maintains a ferroelectric behaviour similar to that of NBT. Further, the estimated off-resonance figure of merit (FOM_off_) for energy harvesting showed an increase of ≈30%, compared to NBT, making the optimised NBNT a promising candidate for multifunctional applications. The literature reports multifunctionality achieved in different solid-solutions which, no doubt provide better properties. Yet processing these materials from simple bulk ceramic compounds to thin-film devices is challenging. Thus, advances in simple perovskite compounds are useful in this respect and this communication aims to show that simple substitution of Nd^3+^ into the A-site of NBT enhances the ferroelectric, piezoelectric, and energy harvesting properties with the added functionality of it being photo-luminescent.

## 2. Experimental details

Polycrystalline NBT compounds with different Nd^3+^ substitutions were synthesized by a conventional solid-state route using high-quality powders of Na_2_CO_3_, Bi_2_O_3_, TiO_2_, and Nd_2_O_3_ (99.99% Sigma-Aldrich Chemicals, USA). Considering the loss on ignition, the precursors are weighed carefully according to stoichiometric ratios given as [Na_0.5_Bi_0.5−*x*_Nd_*x*_]TiO_3_ where *x* = 0, 0.003, 0.005, 0.007, 0.01, 0.02, 0.03, 0.04, 0.05, 0.07, and 0.1) and subsequently milled and calcined at 800 °C for 3 h. The calcined powders are further milled, sieved, and compacted into a circular pellet (*ϕ* = 10 mm) and finally sintered at 1150 °C for 3 h in air with a heating rate of 5°C min^−1^. Phase analysis of the obtained ceramic pellets was performed by using a powder X-ray diffractometer (PANalyticalX'pertpro; CuK_α_ radiation *λ* = 1.5406 Å) over the angles 20° < 2*θ* < 80°. The relative density (RD) of the NBNTs was measured using the Archimedes method and was estimated to be in the range of 94–95% relative to the theoretical density of 5.94 g cm^3^ (ref. 19 and 20) for pure NBT ceramics. Diffused-reflectance measurements were performed using a Shimadzu UV-3600 spectrometer. The room temperature PL spectrum was measured by photo-excitation at 532 nm from a cw-DPSS laser (LSR532NL-300, Lasever) and the subsequent PL was collected by a fiber coupled spectrometer (AvaSpec-ULS2048L-RS, Avantes) with a spectral resolution of 1.4 nm.^21^ The PL spectrum, averaged over 3 spots on the sample, is presented as obtained. All the ceramics are thinned down to a thickness of 0.5 mm to get enough dense silver electrodes on both sides of the pellets. The samples were electrically poled at room temperature in a silicon oil (DOW CORNING 704) bath by applying a DC electric field of (0–10–50) kV cm^−1^ for 30 min and all the measurements were repeated thereafter. The hysteresis (*P*–*E*) data were obtained with a TF-Analyzer 2000 (aixACCT systems, GmbH) from samples of 0.5 mm thickness, using silver-coated electrodes on both sides. The temperature- and frequency-dependent dielectric measurements on the silver-coated pellets were performed with an impedance analyzer (Wayne Kerr 6500B). The piezoelectric harvesting parameters were obtained using a Piezometer System PM300 (Piezotest Ltd.) operated at a frequency of 100 Hz.

## 3. Results and discussion

**Fig. 1:**
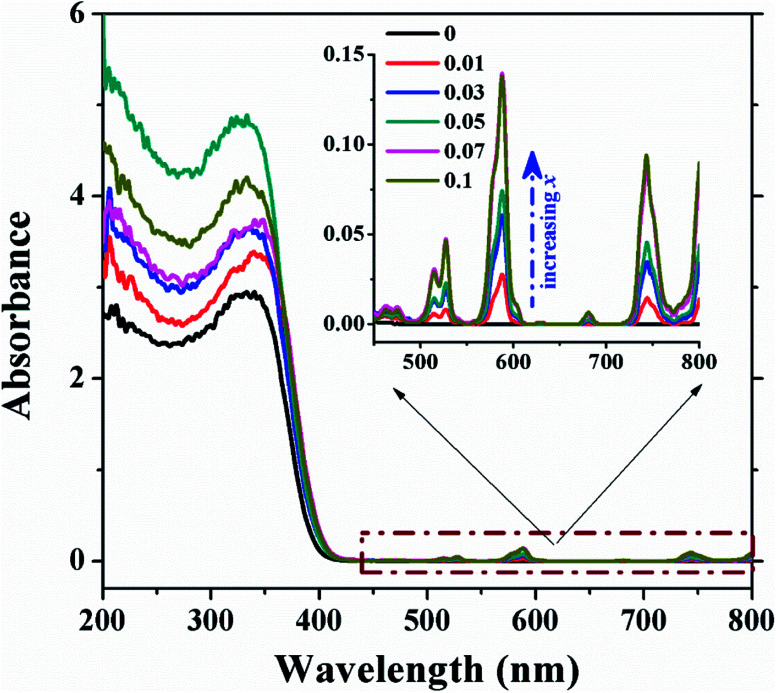
Representative absorption spectra of NBNTs for *x* = 0, 0.01, 0.03, 0.05, 0.07 and 0.1 (inset) showing the magnified 450–800 nm spectral region highlighting the signature absorption of Nd^3+^ ions.

The prepared NBNT ceramics with Nd^3+^ substitution [*x* = 0.003–0.1] allowed a sufficient number of samples to get the optimized NBNT with the best multifunctionality. The absorption spectra of the NBNTs, shown in Fig. 1, had the specific signature of Nd^3+^ in an ordered host medium, even though the peak intensities differ in different hosts.^22^ With an increase in *x* these bands are observed to be getting stronger, with no absorption feature for pure NBT in the visible spectral region (inset).

**Fig. 2:**
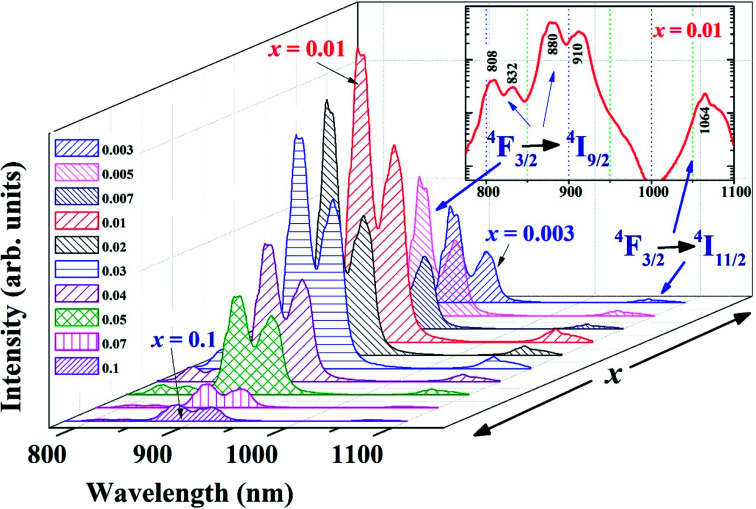
PL spectra of unpoled NBNTs showing the variation in the PL intensity as a function of Nd^3+^ substitution. Inset shows the log-plot of PL spectrum for *x* = 0.01 clearly showing the emission peaks from different transitions.

Fig. 2 shows the measured PL spectra of NBNT ceramics exhibiting multiple peaks within the possible detection range. Evidently, the best PL intensity is observed for *x* = 0.01 substitution and the PL intensity decreases with an increase in the Nd^3+^ substitution. Importantly, the PL peak position is not influenced by *x*. PL is not observed from the pure NBT sample, as expected. The spectroscopic terms related to the radiative relaxation of Nd^3+^ are well known.^22^ The observed PL spectrum consists of overlapping lines arising from transitions between the crystal field split ^4^F_3/2_ level and various sublevels of ^4^I_9/2_ and ^4^I_11/2_. The inset of Fig. 2 shows the PL spectrum from NBNT with *x* = 0.01 substitution, at a log-scale to highlight the various peaks, the maxima being at 880 nm and 910 nm. The choice of rejection color filters at detection enabled observation of the peaks at 808 nm and 832 nm, which were not presented in earlier reports.^14,15^ The measured spectrum in (1000–1100) nm with a maximum at 1064 nm is weak since the detector sensitivity^21^ drops sharply in this spectral region. The shoulder at 1083 nm can be conjectured to be the effect of structural disorder, which could be due to the individual excitation of Nd^3+^ ions in the Bi^3+^ site.

**Fig. 3:**
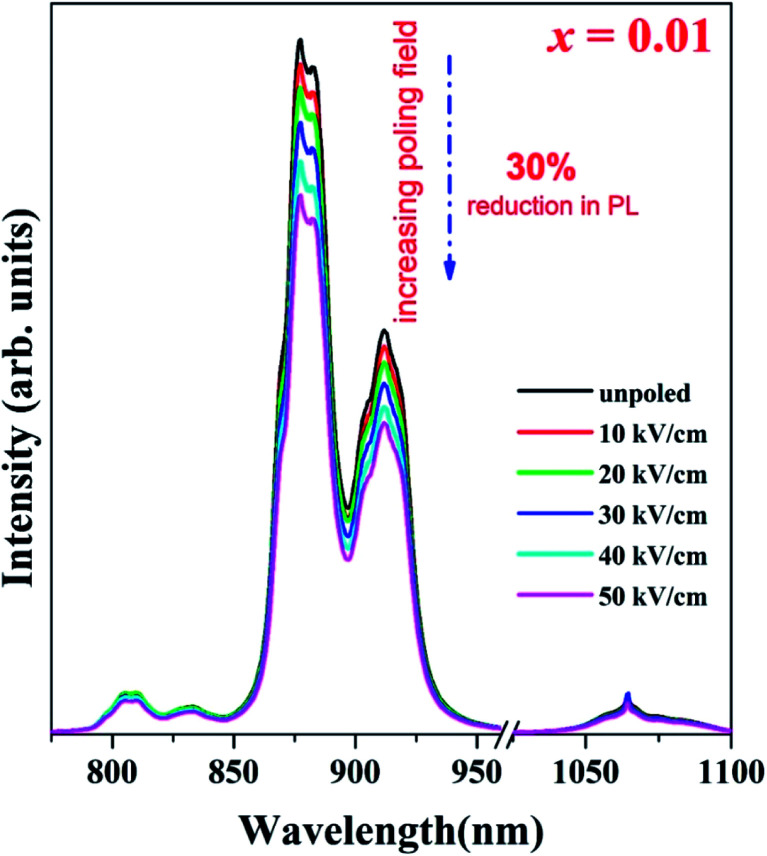
Quenching of PL for optimized NBNT (*x* = 0.01) with different electric fields.

Considering NBNT is a ferroelectric ceramic, it will be subjected to electric fields during routine device operation, making it vital to understand the effect of an applied electric field on the PL. Upon electrical poling, PL is observed to have been quenched in intensity compared to the respective unpoled samples. Nonetheless, the variation in emission with respect to substitution is similar to the trend seen for unpoled ceramics, with the best emission for *x* = 0.01 substitution, similar to emission from unpoled samples. To make a quantitative comparison, we calculated the area under the PL spectra of poled and unpoled ceramics in the spectral range of (800–1000) nm. Fig. 3 demonstrates the effect of electrical poling, with a reduction in PL spectral intensity of ≈30% upon poling to 50 kV cm^−1^ being observed. It is interesting to note that the shapes of the PL spectral profiles remain unchanged upon poling, unlike a similar report^12^ on Eu^3+^ doped NBT ceramics for which the formation of a new spectral peak was observed. The PL intensity is found to be a maximum for *x* = 0.01 substitution.

Considering the paucity of reports on the computational study of crystal-field effects on rare-earth emission in doped perovskite material systems, we try to elucidate our observation qualitatively. It is well known that the Nd^3+^ emission is extremely sensitive to the local host symmetry. The Judd–Ofelt theory, which is widely used to estimate the intensities of transitions for the RE ions, defines a set of three intensity parameters *Ω*_*t*_ (*t* = 2, 4 and 6) that are sensitive to the local environment of the luminescent centers.^23,24^ Here, the important factors affecting the local environments of Nd^3+^ in NBT pertain to fluctuations in the Na : Bi ratio from 1 : 1 as a result of substitution, different chemical ordering at the local scale and disorder associated with atomic displacements. With a solid matrix with fixed lattice positions for Na and Bi, the external electric field is expected to induce long-range order in the polar displacements of the atoms. This in turn affects the transition intensities. Therefore, in line with an earlier report^16^ for Pr-doped BNT-BT, where there was an enhancement in PL intensity upon poling, the observed reduction in PL can be conjectured to be a result of electric-field induced structural order towards higher symmetry.

**Fig. 4:**
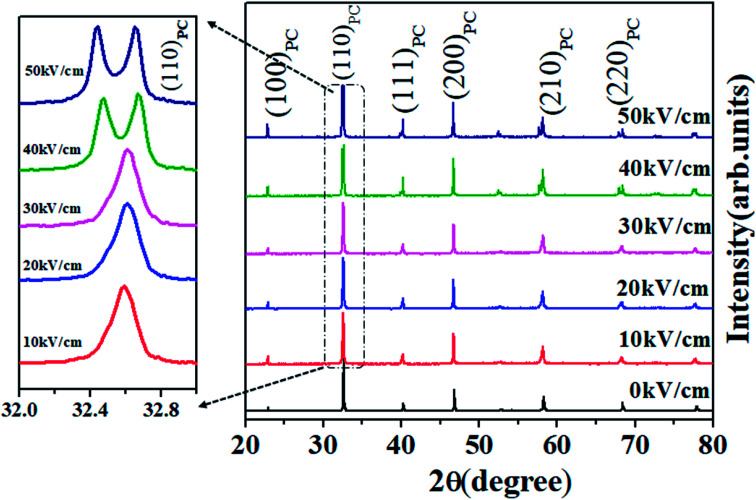
X-Ray diffraction pattern of NBT-0.01Nd under different poling fields (0–10–50) kV cm^−1^, showing the enlarged portion of (110)_PC_.

To confirm the role of structural ordering, XRD measurements were performed in different poling fields (0–50) kV cm^−1^ with the interval of the poling field 10 kV cm^−1^ to identify the structural symmetry variation in the optimal specimen, *i.e.* NBT-0.01Nd. Unpoled NBNTs exhibited a single phase structure stabilizing into a monoclinic structure with *Cc* space group symmetry in the substitution range 0 ≤ *x* ≤ 0.1, with no co-existing secondary phases within the sensitivity of XRD.^22^Fig. 4 shows the variation in the XRD pattern for various poling fields. The poling field induces a distinct broadness in the main prominent peak (110)_PC_ of the perovskite structure, as shown in the left panel of Fig. 4. Notably, the structurally induced changes we observe upon electrical poling are in line with the reduction in PL intensity presented in Fig. 3.

**Fig. 5:**
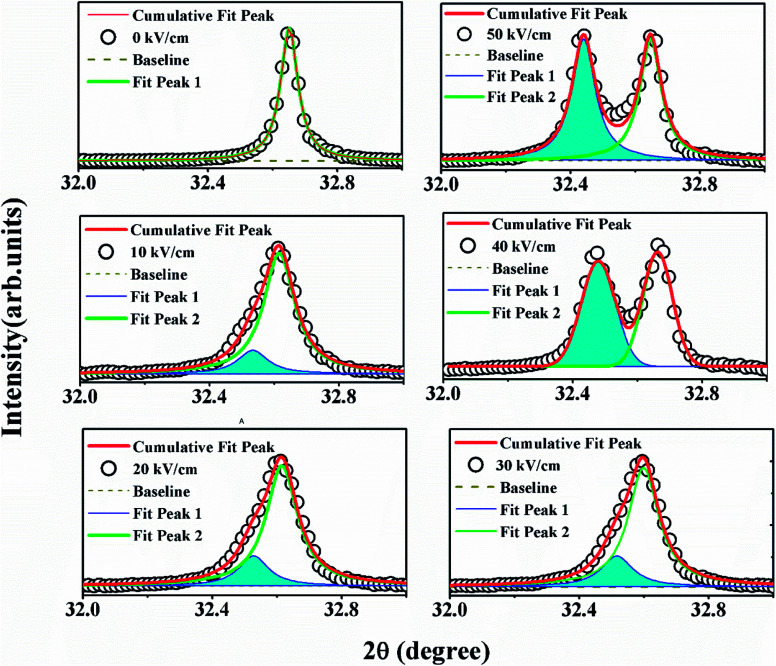
Deconvolution of the (110)_PC_ peak for extracting the rhombohedral *R*3*c* phase along with monoclinic *Cc* under different electrical poling fields (0–50) kV cm^−1^.

Further analysis of this prominent peak presented the structural evolution of other phases from the peak broadening as well the change in peak profile. In order to extract the possible structural evolution under the poling field in (110)_PC_, the obtained peak is deconvoluted by using Lorentzian multi-peak fits, as shown in Fig. 5. The new structural phase rhombohedral (*R*3*c*) gradually evolves over the poling fields (represented by the blue shaded portion of the area under the curve). From the 40 kV cm^−1^ spectrum we can observe a prominent doublet instead of a singlet, representing the effect of electrical poling on the crystal structure. The XRD data showing the effect of poling throughout the series is provided in the ESI.†

**Fig. 6:**
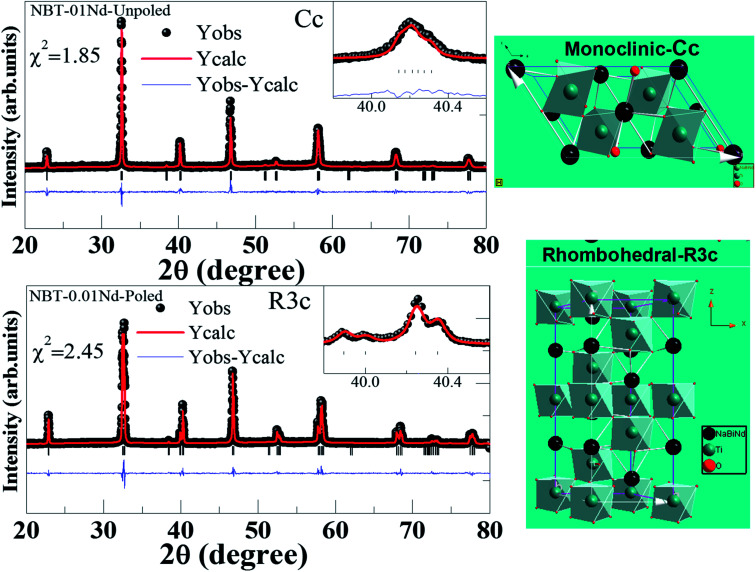
(Top panel) The structural refinement of unpoled NBT-0.01Nd with monoclinic *Cc* and corresponding crystal structure. (Bottom panel) The structural refinement of poled NBT-0.01Nd with rhombohedral *R*3*c* and corresponding crystal structure.

To strengthen the above arguments, further structural refinement of the XRD measurements was carried out by Rietveld refinement for the poled and unpoled [Na_0.5_Bi_0.5−*x*_Nd_*x*_]TiO_3_ (*x* = 0.01) using Fullproof software, as presented in Fig. 6. Notably, the shape of the Bragg profile was modified upon poling, suggesting a gradual change throughout the series [Na_0.5_Bi_0.5−*x*_Nd_*x*_]TiO_3_ (*x* = 0 and 0.1). The structure throughout the series stabilizes to monoclinic with the *Cc* space group. Upon poling the induced structural modification from *Cc* to *R*3*c* changes, observed as clear splitting at the (110)_PC_ reflection. The unpoled XRD pattern of [Na_0.5_Bi_0.5−*x*_Nd_*x*_]TiO_3_ (*x* = 0.01) has fitted reasonably well with the recently proposed monoclinic *Cc* structure. The poled pattern of the same composition could be fitted well with the pure rhombohedral (*R*3*c*) structure. The goodness of fit is depicted in the inset of Fig. 6 for the Bragg reflection at 40°. The right panel of Fig. 6 illustrates the proposed crystal structure corresponding to poled and unpoled ceramics, using the refined parameters as input to the Diamond-Crystal and Molecular Structure Visualization software. Evidently distinct structural modulation can be observed upon poling. Further, the XRD patterns of the poled compositions *x* = 0.04 onwards could be fitted well with the *Cc* model, showing a reduced contribution of the fraction of the *R*3*c* phase upon poling the compounds with increased Nd^3+^ substitution. Table 1 shows the refined crystal parameters and corresponding positions.

Refined parameters and atomic positions of monoclinic *Cc* and rhombohedral *R*3*c* of NBT-0.01Nd

**Table:** 

Unpoled (*Cc*)	*x*	*y*	*z*
Na/Bi/Nd	0.0000	0.2517	0.0000
Ti	0.2519	0.2504	0.7386
O_1_	−0.0016	0.2521	0.5107
O_2_	0.1982	0.4906	−0.0686
O_3_	0.3013	0.0296	0.0614
Lattice parameters (Å)	*a* = 9.518, *b* = 5.4876, *c* = 5.4982, *α* = *γ* = 90°, *β* = 125.331°, *χ*^2^ = 1.85

**Table:** 

Poled (*R*3*c*)	*x*	*y*	*z*
Na/Bi/Nd	0.0000	0.0000	0.2713
Ti	0.0000	0.0000	0.0068
O	0.1335	0.3379	0.0838
Lattice parameters (Å)	*a* = 5.4789, *b* = 5.4789, *c* = 13.5465, *α* = *β* = 90°, *γ* = 120°, *χ*^2^ = 2.45		

**Fig. 7:**
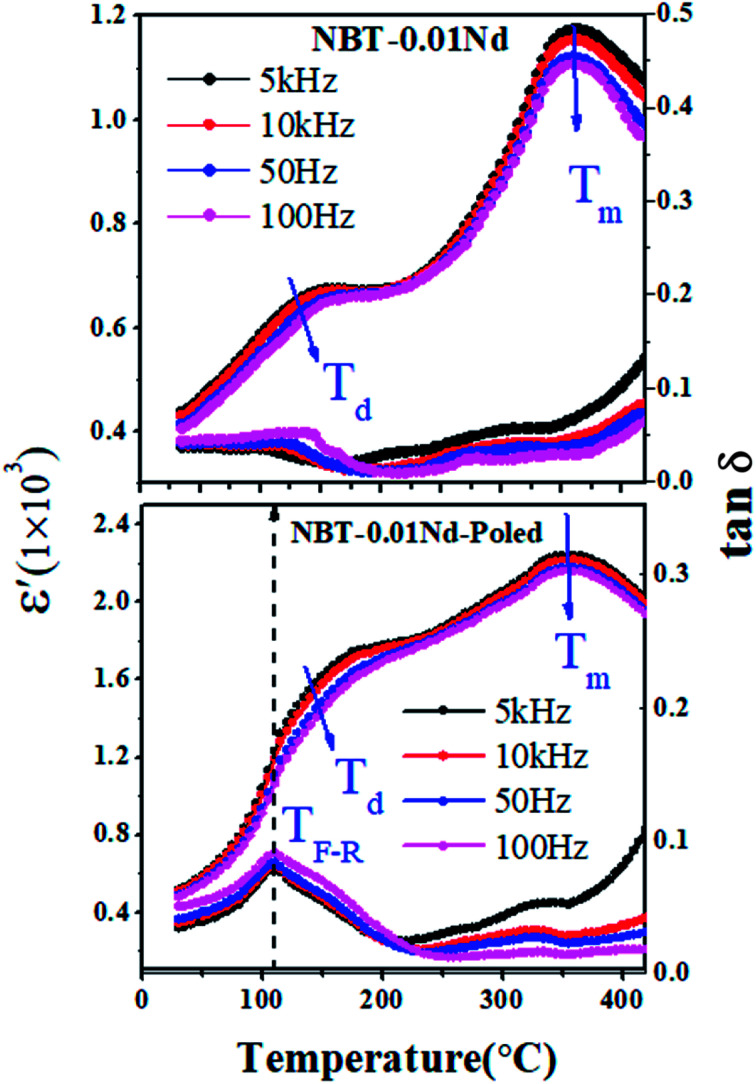
The dielectric constant and tangent loss with temperature of NBT-0.01Nd for (top) unpoled and (bottom) poled samples.

In order to understand the difference in the state of dipolar ordering in unpoled and poled specimens of NBT-0.01Nd, we performed temperature-dependent studies of the dielectric constant and loss tangent (tan *δ*) at different frequencies within the range of 5–100 kHz, as shown in Fig. 7. There are two prominent dielectric anomalies, corresponding to the temperatures *T*_d_ (the depolarization temperature) and *T*_m_ (the maximum temperature), which are responsible for the phase transitions from ferroelectric to antiferroelectric and antiferroelectric to paraelectric over the temperature range. The *T*_d_ and *T*_m_ were found to be 140 °C and 360 °C, respectively, from the poled and unpoled samples. In the case of the poled specimen, the dielectric constant improved compared to the unpoled specimen and a strong frequency dispersion with frequency dependence was observed at broad maxima of *T*_d_ and *T*_m_. After poling, a new dielectric anomaly, which is associated with the transition from ferroelectric to relaxor and antiferroelectric relaxor at *T*_(F–R)_, can be observed clearly from the dielectric and loss curve at 110 °C in the poled specimen.

The PL intensity variation and subsequent PL quenching with higher Nd^3+^ substitution can be understood by invoking concentration quenching.^25,26,27^ Theoretically, the rate of relaxation due to concentration quenching varies as *r*^−6^ (where *r* denotes the inter-ion distance). As the concentration increases, the distance between neighboring Nd^3+^ ions reduces, leading to an energy exchange among Nd^3+^ ions. The inter-ionic radius for different *x* is estimated by using the relation.^28^
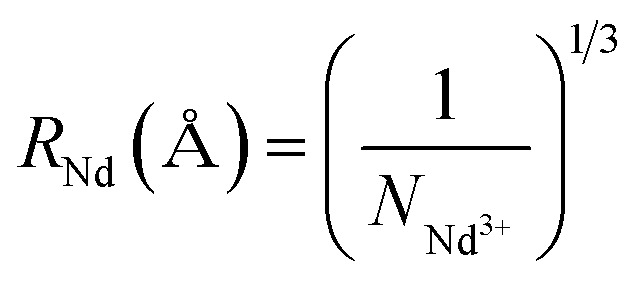
 Here, *N*_Nd_^3+^ is the Nd^3+^ concentration in ions per cm^3^. As expected, an increase in *x* leads to a reduction in *R*_Nd_. For the unpoled NBNT with *x* = 0.01, the estimated *R*_Nd_ ≈ (23.2 ± 1.3) Å. Similar calculations can be made for the poled NBNTs. From Fig. 2, we deduce the critical concentration *X*_Critical_ = 0.01, after which concentration quenching by energy transfer begins. The theoretical transfer distance, *R*_C_ (defined as the distance for which the energy transfer probability between neighboring Nd^3+^ ions is equal to the emission probability of the Nd^3+^ ions) is estimated using the relation^29^
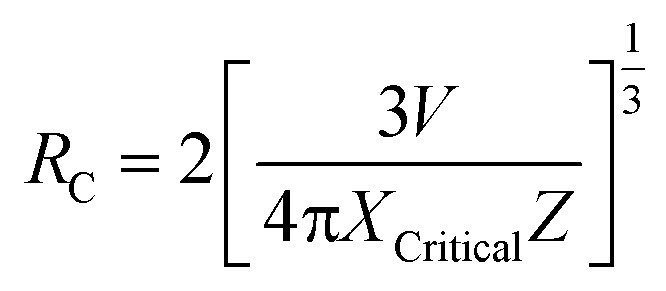
, where *V* is the volume of one unit cell, *X*_Critical_ is the optimal concentration of the activator ion and *Z* is the number of cations present in the unit cell. Using the values, *V* = 234.1 Å^3^, *Z* = 4 for a monoclinic structure (*Cc*, unpoled), we estimated *R*_C_ ≈ (22.3639 ± 1.5) Å which is in the vicinity of the value of *R*_Nd_ for *x* = 0.01. Similarly, for the poled compound, using *V* = 351.5 Å^3^, *Z* = 6 for the rhombohedral (*R*3*c*) structure, and we estimated *R*_C_ ≈ (22.3612 ± 1.5) Å, which is similar to the value for the unpoled structure and in the vicinity of the value of *R*_Nd_ for *x* = 0.01. From our calculations we deduce that the critical energy transfer radius (*R*_C_) and the inter-ionic radius (*R*_Nd_) are equal within the error limits for *x* = 0.01 substitution and beyond this concentration quenching begins.

**Fig. 8:**
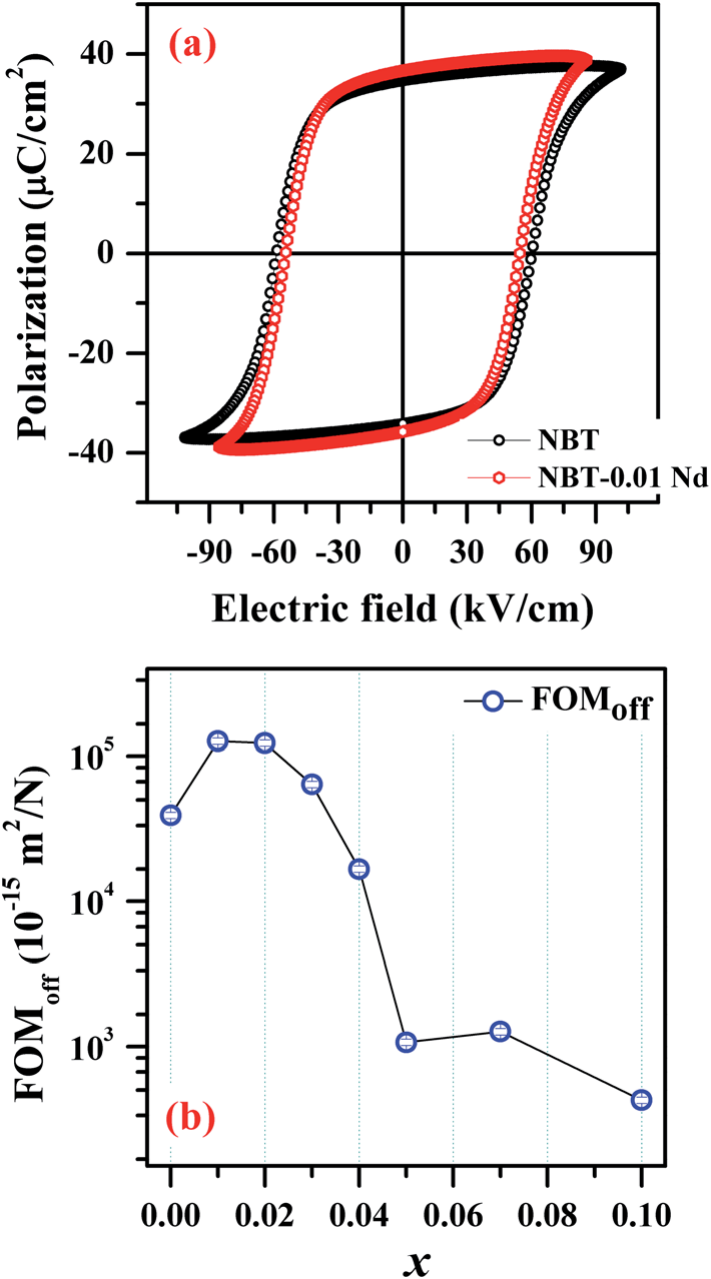
(a) Room temperature polarization (*P*)–electric field (*E*) measurement of NBT and NBT-0.01Nd. (b) Plot showing the variation of FOM_off_ as a function of *x* (Nd^3+^ substitution).

To achieve multifunctionality, it is vital to have this PL functionality without compromising on the existing ferroelectric nature of the parent NBT compound. Fig. 8(a) presents a comparison of the hysteresis (*P*–*E*) loops obtained for the parent NBT and the optimized NBNT (*x* = 0.01). These hysteresis loops are well saturated, which is characteristic of ferroelectric behavior. We performed the measurement on two devices of each composition and estimated the average coercive electric fields (*E*_c_) to be ≈65 kV cm^−1^ and 59 kV cm^−1^ and the remnant polarization values (*P*_r_) to be 32.7 μC cm^−2^ and 33.8 μC cm^−2^ for NBT and NBT-0.01Nd, respectively. NBT-0.01Nd exhibits a slightly higher *P*_r_ and much lower *E*_c_ values by 3% and 12%, respectively, relative to pure NBT, confirming the better suitability of NBNT for possible applications in piezochromic devices. The requirement for such NBT based devices is lower coercivity (for easy switching) without compromising *P*_r_. Thus, it can be inferred that the NBT-0.01Nd compound can be considered as a possible candidate for optimizing future piezochromic devices.

The best enhancement in terms of device functionality upon substitution was observed for piezoelectric energy harvesting. The measured *d*_33_ were 84 pC N^−1^ and 95 pC N^−1^ for NBT and NBT-0.01Nd, suggesting an increase of 13%, implying that substitution of Nd^3+^ in NBT favors the mobility of domain walls (corroborated by the softer ferroelectric nature). From the same measurements we estimated the piezoelectric voltage coefficient (*g*_33_) to be 26.7 mV N^−1^, 48.4 mV N^−1^ and the dielectric loss (tan *δ*) to be 0.0572, 0.0362 for NBT and NBT-0.01Nd, respectively. Together with the three quantities estimated so far, the off-resonance figure of merit (FOM_off_) for energy harvesting can be estimated using the relation
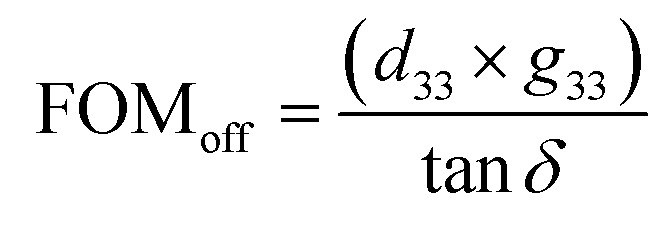
.^30,31^ The estimated values of FOM_off_ were 39 209.8 × 10^−15^ m^2^ N^−1^ and 127 016.6 × 10^−15^ m^2^ N^−1^ for NBT and NBNT, implying an enhancement of ≈30%. Fig. 8(b) shows the variation in FOM_off_ for all the various composition used in the study. Interestingly, the variation in FOM_off_ follows the trend in PL emission for the varying amounts of Nd^3+^ substituted in NBT.

## 4. Conclusions

To conclude, we presented a comprehensive investigation of the effect of Nd^3+^ in Na_0.5_Bi_0.5−*x*_Nd_*x*_TiO_3_ (NBNT) in order to obtain various functionalities. The optimal concentration of Nd^3+^ for the best PL emission was found to be *x* = 0.01 (≅1 mol%). Upon electrical poling, there was a significant quenching of PL intensity. We elucidated this to be a result of electric-field induced structural ordering to higher symmetry. Structural studies from XRD data corroborated this hypothesis, as the electrical poling resulted in the transformation of the stabilized NBNT phase from a monoclinic to a rhombohedral structure which has higher symmetry. Estimation of the critical radius (*R*_C_) for energy transfer and inter-ionic distance (*R*_Nd_) suggested the onset of a concentration quenching effect after the substitution of *x* = 0.01. For the optimized NBNT the electrical characterization revealed a reduction in the coercive electric field (*E*_c_) by 12% compared to undoped NBT. The piezoelectric characterization of the samples studied showed an enhanced *d*_33_coefficient for the optimized NBNT and therefore the estimated off-resonance figure of merit (FOM_off_) for energy harvesting revealed ≈30% enhancement for NBT-0.01Nd. From a computational knowledge point of view, the effect of perovskite structure symmetry on Nd^3+^ emission has not been studied to date. Thus, we hope our results will encourage such investigations as the very possibility of having multifunctionality in a simple material system is elegant. The results presented here convey a simple material system, but with elegant multifunctional properties. Therefore, the optimally substituted NBNT displayed convincing reasons for it to be a potential multifunctional material exhibiting simultaneous ferroelectric, luminescent and energy harvesting properties.

## Conflicts of interest

There are no conflicts to declare.

## Supplementary Material

RA-008-C8RA01349G-s001
