# Supplementary material for: Multifunctional Nd3+ substituted Na0.5Bi0.5TiO3 as lead-free ceramics with enhanced luminescence, ferroelectric and energy harvesting properties
Source: RSC Adv. 2018 Apr 23;8(28):15282–9. doi: 10.1039/c8ra01349g (PMC9080032; doi:10.1039/c8ra01349g)
Supplement: RA-008-C8RA01349G-s001 [file RA-008-C8RA01349G-s001.pdf]

Electronic Supplementary Material (ESI) for RSC Advances.
This journal is © The Royal Society of Chemistry 2018

Multifunctional Nd^{3+} substituted $Na_{0.5}Bi_{0.5}TiO_3$ as lead-free ceramics with enhanced luminescence, ferroelectric and energy harvesting properties

Kumara Raja Kandula, Saket Asthana[†] and Sai Santosh Kumar Raavi[*]

Advanced Functional Materials Laboratory, Department of Physics, Indian Institute of Technology Hyderabad, Kandi, Sangareddy 502285, Telangana, India

[*]sskraavi@iith.ac.in , [†]asthanas@iith.ac.in

Structural information (#)

Figure 1 shows the comparison of $(110)_{pc}$ peaks obtained from the XRD data of all samples for unpoled and poled (@50kV/cm) samples. The unpoled samples stabilize into monoclinic structure with Cc space group symmetry,[1] as shown in the left panel, which is in good agreement with the JCPDS card no: 01-080-4260. In case of unpoled sample the gradual shift of $(110)_{pc}$ peak towards a higher Bragg angle evolution with increasing x. Considering that Nd^{3+} has smaller ionic radius (1.27Å,12-coordinates) compared to Bi^{3+} (1.37Å,12-coordinates), gradual substitution of Nd^{3+} in place of Bi^{3+} in the NBT matrix, leads gradual contraction of the unit cell volume due to reduction in A-site average ionic radii. The electrical poling of these ceramics immediately stabilizes into rhombhohedral crystal structure with $R3c$ space group until ($0 < x < 0.03$), which is good agreement with the JCPDS card no: 01-070-9850. Beyond $x = 0.03$ substitution (i.e, $0.04 < x < 0.1$), the XRD data show formation of mixed phase with symmetry resembling $Cc + R3c$ space group as shown in the right panel of Fig 7. Thus, the XRD investigation reveal the electrical poling, induces structural order and the NBNT transform from the lower symmetry (Cc) to a higher symmetry ($R3c$) resulting in the change in the local host environment and consequently quenching PL intensity.

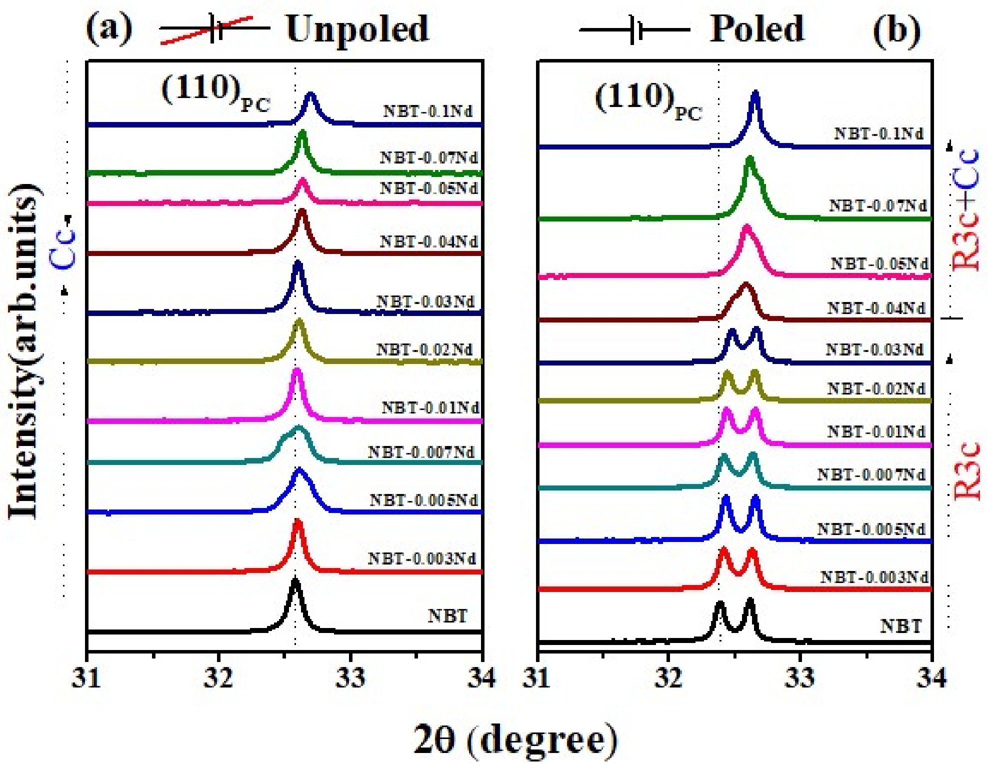

Fig.1. (a) The unpoled XRD pattern of Bragg reflection $(110)_{pc}$ shows the monoclinic Cc Space group shown. (b) The poled XRD pattern of Bragg reflection $(110)_{pc}$ shows the rhombohedral $R3c$ and retain to Cc after ($x > 0.04$) shown.

References:

1. K. Kandula, S. S. K. Raavi & S. Asthana, *J. Alloy. Comp*, 2018, 732, 233-239.
